# Human Monoclonal Antibody 81.39a Effectively Neutralizes Emerging Influenza A Viruses of Group 1 and 2 Hemagglutinins

**DOI:** 10.1128/JVI.01284-16

**Published:** 2016-11-14

**Authors:** Henju Marjuki, Vasiliy P. Mishin, Ning Chai, Man-Wah Tan, Elizabeth M. Newton, John Tegeris, Karl Erlandson, Melissa Willis, Joyce Jones, Todd Davis, James Stevens, Larisa V. Gubareva

**Affiliations:** a Influenza Division, National Center for Immunization and Respiratory Diseases, Centers for Disease Control and Prevention, Atlanta, Georgia, USA; b Infectious Diseases, Genentech Inc., South San Francisco, California, USA; c Portfolio Management and Operations Departments, Genentech Inc., South San Francisco, California, USA; d Influenza Division, Biomedical Advanced Research and Development Authority, Washington, DC, USA; Icahn School of Medicine at Mount Sinai

## Abstract

The pandemic threat posed by emerging zoonotic influenza A viruses necessitates development of antiviral agents effective against various antigenic subtypes. Human monoclonal antibody (hMAb) targeting the hemagglutinin (HA) stalk offers a promising approach to control influenza virus infections. Here, we investigated the ability of the hMAb 81.39a to inhibit *in vitro* replication of human and zoonotic viruses, representing 16 HA subtypes. The majority of viruses were effectively neutralized by 81.39a at a 50% effective concentration (EC_50_) of <0.01 to 4.9 μg/ml. Among group 2 HA viruses tested, a single A(H7N9) virus was not neutralized at 50 μg/ml; it contained HA_2_-Asp19Gly, an amino acid position previously associated with resistance to neutralization by the group 2 HA-neutralizing MAb CR8020. Notably, among group 1 HA viruses, H11-H13 and H16 subtypes were not neutralized at 50 μg/ml; they shared the substitution HA_2_-Asp19Asn/Ala. Conversely, H9 viruses harboring HA_2_-Asp19Ala were fully susceptible to neutralization. Therefore, amino acid variance at HA_2_-Asp19 has subtype-specific adverse effects on *in vitro* neutralization. Mice given a single injection (15 or 45 mg/kg of body weight) at 24 or 48 h after infection with recently emerged A(H5N2), A(H5N8), A(H6N1), or A(H7N9) viruses were protected from mortality and showed drastically reduced lung viral titers. Furthermore, 81.39a protected mice infected with A(H7N9) harboring HA_2_-Asp19Gly, although the antiviral effect was lessened. A(H1N1)pdm09-infected ferrets receiving a single dose (25 mg/kg) had reduced viral titers and showed less lung tissue injury, despite 24- to 72-h-delayed treatment. Taken together, this study provides experimental evidence for the therapeutic potential of 81.39a against diverse influenza A viruses.

**IMPORTANCE** Zoonotic influenza viruses, such as A(H5N1) and A(H7N9) subtypes, have caused severe disease and deaths in humans, raising public health concerns. Development of novel anti-influenza therapeutics with a broad spectrum of activity against various subtypes is necessary to mitigate disease severity. Here, we demonstrate that the hemagglutinin (HA) stalk-targeting human monoclonal antibody 81.39a effectively neutralized the majority of influenza A viruses tested, representing 16 HA subtypes. Furthermore, delayed treatment with 81.39a significantly suppressed virus replication in the lungs, prevented dramatic body weight loss, and increased survival rates of mice infected with A(H5Nx), A(H6N1), or A(H7N9) viruses. When tested in ferrets, delayed 81.39a treatment reduced viral titers, particularly in the lower respiratory tract, and substantially alleviated disease symptoms associated with severe A(H1N1)pdm09 influenza. Collectively, our data demonstrated the effectiveness of 81.39a against both seasonal and emerging influenza A viruses.

## INTRODUCTION

Influenza A viruses of 16 hemagglutinin (HA) and nine neuraminidase (NA) antigenic subtypes have been detected in a vast natural reservoir of aquatic birds. Eight highly diverse HA subtypes are known to have caused infections in humans (H1, H2, H3, H5, H6, H7, H9, and H10); some of these subtypes (H1, H2, and H3) have caused pandemics and recurrent epidemics with various levels of severity. Interspecies transmission and zoonosis are central to the emergence of new viruses in humans. For example, a swine-origin virus contributed to the genesis of the 2009 H1N1 pandemic [A(H1N1)pdm09] and continues to circulate among humans (1). Since 2013, China has experienced four waves of outbreaks caused by avian A(H7N9) viruses despite significant control measures. More recently, subclade 2.3.4.4 highly pathogenic avian influenza (HPAI) H5 viruses were detected in several countries in Europe and Southeast Asia (2) and were detected for the first time in wild birds and poultry in North America, where they inflicted a significant economic burden on the poultry industry (2) and raised concerns over potential human infections. Furthermore, in January 2016, both HPAI and low-pathogenicity avian influenza (LPAI) A(H7N8) viruses were detected in turkey flocks in Indiana, USA. As with HPAI virus of the H5 subtype, this is the first instance of HPAI A(H7N8) virus detection in poultry, whereas LPAI A(H7N8) virus has been detected previously in wild bird surveillance in the U.S.

Antiviral therapy has been a valuable component of the ongoing efforts to reduce the burden of disease caused by influenza virus infections in humans. Antiviral agents have the potential to provide a key defense at times when vaccination is not effective at preventing infection in individuals or is not available. The current treatment options in the U.S. are limited to M2 protein blockers and NA inhibitors (NAIs). These two classes of influenza drugs are represented by small molecules that target highly conserved regions of two viral surface proteins, a transmembrane domain of M2 and a catalytic site of NA. The NAIs oseltamivir (oral) and zanamivir (inhaled) were the first two FDA-approved antivirals for treating influenza infections, with oseltamivir being the most widely prescribed. Intravenous peramivir has also recently been FDA approved, while intravenous zanamivir is undergoing clinical evaluation but has been available for compassionate use in patients with serious influenza-related illness (3). The intravenous route of drug administration is viewed as the most convenient in treating severely ill patients.

Resistance to M2 blockers is widely present among seasonal influenza A viruses, and the U.S. Centers for Disease Control and Prevention (CDC) has not recommended their use since the 2005-2006 influenza season (4). Moreover, many HPAI H5N1 and swine-origin viruses are also M2 resistant (5, 6). Resistance to NAIs, especially oseltamivir, has been detected in viruses following patient treatment (7, 8), and widespread oseltamivir resistance occurred in seasonal A(H1N1) viruses prior to the emergence of A(H1N1)pdm09 (9, 10). These drugs should be administered early after viral infection to achieve the optimal therapeutic benefits (11, 12). Concerns of widespread resistance, reported side effects, and the less than desirable potency of NAIs emphasize the need to expand therapeutic options, especially for the most vulnerable patient populations. Antiviral agents with new mechanisms of action that are also efficacious against a broad spectrum of influenza virus subtypes are preferred. Therefore, efforts to find antivirals that interfere with HA protein function are desirable despite the genetic/structural diversity and continuous evolution of this protein. To this end, the development of human monoclonal antibodies (MAbs) with a broad spectrum of anti-HA activity is promising.

The 16 HA subtypes are divided into groups 1 and 2 based on their phylogenetic relationship: group 1 encompasses H1, H2, H5, H6, H8, H9, H11, H12, H13, and H16, while group 2 includes H3, H4, H7, H10, H14, and H15 (13). The influenza virus HA is a trimeric type I transmembrane glycoprotein formed by identical HA_0_ molecules, which are cleaved by host proteases into HA_1_ and HA_2_ subunits (14). The HA_1_ subunit forms a membrane-distal globular head that contains the receptor-binding site and most of the highly variable immunodominant antigenic regions recognized by neutralizing antibodies (15, 16). These antibodies are typically strain or subtype specific. Conversely, the HA_2_ subunit consists of a highly conserved stalk-like structure, which anchors the globular domain to the viral membrane (17, 18). HA_2_ contributes to the stalk region that carries out the membrane fusion function; generally, antibodies raised to this highly conserved region are broadly neutralizing. While broadly neutralizing antibodies (bnAbs) represent only a small fraction of the antibodies raised (19, 20), their discovery has expanded interest in bnAbs as potential antiviral drugs.

Further advancement has been achieved with discoveries of fully human MAbs that neutralize viruses of both HA groups. These MAbs are 39.29 and 81.39 (21); FI6v3 (22); CR9114 (23); PN-SIA-28 (24); CT149 (25); VIS410 (26, 27); 045-051310-2B06 and S6-B01 (28); 05-2G02 (29); 07-5E01, 41-5D06, and 41-5E04 (30); HV6-1+HD3-3, HV1-18+HD3-9, and HV1-18 (Q-x-x-V) (31); and MEDI8852 (32). Nonetheless, their antiviral activities against all known subtypes of influenza A viruses has not been completely characterized. While the *in vivo* effectiveness of these broadly neutralizing antibodies has been tested using mouse (e.g., for MAbs CR9114, CT149, FI6v3, 39.29, and VIS410) and ferret (e.g., for MAbs FI6, 39.29, 81.39) models, studies with additional challenge viruses are warranted.

Nakamura et al. described a human-plasmablast enrichment technique that allowed the identification of two stalk-targeting MAbs, 39.29 and 81.39, with neutralizing activity against A(H1N1) (group 1) and A(H3N2) (group 2) influenza viruses *in vitro*. The structural analysis showed that 39.29 and 81.39 MAbs utilized the same germ lines, with each containing 12 to 15 variable heavy-chain and 7 to 9 variable light-chain mutations (21). The therapeutic effect of 39.29 was tested against A(H1N1) (group 1) and A(H3N2) (group 2) subtypes in mice, while both 39.29 and 81.39 were tested in ferrets infected with an HPAI A(H5N1) (group 1) virus. A single-dose administration of either MAb in a postexposure treatment model was superior to repetitive treatment with oseltamivir (21). However, the neutralization activity of these MAbs against other influenza subtypes has not been elucidated, and their efficacy against newly emerging zoonotic influenza viruses is not known.

Here, we show that human IgG1 MAb 81.39a, a derivative of the parental 81.39, exerts a broadly neutralizing activity against a large panel of influenza A viruses covering 16 HA subtypes, including the emerging clade 2.3.4.4 HPAI H5Nx, HPAI A(H7N8), and A(H7N9) viruses. Here, we observed differences in 81.39a neutralization profiles and provide the molecular basis of 81.39a activity *in vitro*. Furthermore, we demonstrated that a single administration of 81.39a protected mice and ferrets against lethal challenge with A(H1N1)pdm09, HPAI A(H5N2), HPAI A(H5N8), A(H6N1), and A(H7N9) influenza viruses. These studies provide further insight into the breadth of the antiviral activity of 81.39a and expand our understanding of this stalk-targeting MAb as a promising therapeutic option against seasonal and emerging influenza viruses.

## MATERIALS AND METHODS

### Ethics statements.

Animal experiments were conducted in strict compliance with guidelines of the CDC Institutional Animal Care and Use Committee in association with the PHS Policy, the Animal Welfare Act (USDA), and the *Guide for Animal Care and Use of Laboratory Animals* (33). Animal protocols were approved by the CDC IACUC committee. Animal welfare was observed on a daily basis until study completion. All procedures involving zoonotic influenza viruses were performed under animal biosafety level 3 enhanced (ABSL3+) conditions and in an ABSL2 facility when working with seasonal viruses. Manipulation of animals and collection of organ tissues were conducted within a biosafety cabinet.

### Viruses and sequence analysis.

Seasonal and zoonotic influenza viruses used in this study were submitted to the WHO Collaborating Center for Surveillance, Epidemiology, and Control of Influenza at the Centers for Disease Control and Prevention (CDC) for virological surveillance, which includes antigenic characterization and antiviral susceptibility testing. Non-U.S. viruses were kindly shared with the CDC by the responsible authorities in designated countries. Two A(H7N9) clones of A/Taiwan/1/2013 virus used in this study were isolated using biological cloning (plaque purification) procedures. All viruses were propagated in Madin-Darby canine kidney (MDCK) cells (ATCC, VA) and underwent a limited number of passages in cultured cells to maintain their original properties. At least viral HA and NA gene sequences were confirmed by Sanger sequencing generated using an ABI Prism 3730 genetic analyzer (Applied Biosystems, Foster City, CA) and assembled using Sequencher 5.0 (Gene Codes Corporation, Ann Arbor, MI). The amino acid substitution(s) was corroborated using the pyrosequencing method as previously described (34, 35). Protein sequences for viral HA of H9, H12, and other virus subtypes used for HA_2_ sequence analyses were downloaded from the public database Global Initiative on Sharing All Influenza Data (GISAID).

### Human monoclonal antibody.

The broadly neutralizing human 81.39a (IgG1) MAb for influenza A viruses was generated, purified, and produced in large quantity as previously described (21). Anti-influenza B virus MAb was used as a negative control in focus and virus yield reduction assays. Control IgG (gD5237), a MAb specific for glycoprotein D of herpes simplex virus (21), was applied for all *in vivo* studies.

### Virus neutralization and inhibition of viral replication in cell culture.

The neutralization properties of 81.39a against 16 virus subtypes were examined using the focus reduction assay. Briefly, confluent MDCK-SIAT1 cell monolayers in 24-well plates were incubated with a mixture of 5-fold serial dilutions of MAb at 0.08 to 500 μg/ml and pretitrated virus (30 to 60 foci/well), which was allowed to react for 1 h at room temperature, for 1 h at 37°C. After removal of virus-MAb mixture, overlay (0.5% Avicel in 1× Eagle's modified essential medium [EMEM]; ATCC, VA) containing MAb was added to the cells, and they were further incubated for 16 to 20 h at 37°C. Overlay then was removed, and cells were washed and fixed. Visualization of infected cells was done by immunostaining with mouse anti-viral nucleoprotein (NP) antibody blend (MAB8251; Millipore, CA), followed by incubation with anti-mouse IgG (Fab specific)-fluorescein isothiocyanate (FITC) antibody (Sigma, MO). The number of foci (a focus is a cluster of five or more infected cells) was counted under a fluorescence microscope.

A virus yield reduction assay was applied to assess the inhibitory effect of 81.39a on virus replication in MDCK-SIAT1 cells. After 1 h of inoculation at 37°C as described above, the inoculum was replaced with fresh cell culture medium containing MAb, and cells were incubated for 24 h at 37°C. Thereafter, supernatants were collected and used for virus titration using the 50% tissue culture infectious dose (TCID_50_) assay.

### Infection, MAb treatment, and collection of tissue samples in mice.

Six- to 8-week-old female BALB/c mice (Charles River, MA) were housed and monitored for at least 3 days for acclimation prior to the start of the study. Groups of 22 mice were inoculated with 10^5.6^ 50% egg infectious doses (EID_50_) (∼4.0 50% mouse lethal doses [MLD_50_]) of H5N2 virus or 10^6.6^ EID_50_ (∼1.6 MLD_50_) of A(H5N8) virus. Mice were then treated with either 81.39a (45 mg/kg of body weight) or control IgG (gD5237; 45 mg/kg) at 24 days postinoculation (dpi) via intravenous (tail vein) injection (50 μl/mouse) under anesthesia. Lungs (*n* = 12/treatment group) were collected at days 3 and 6 (6 mice per test day) as previously described (36).

In the second set of experiments, groups of 14 mice were inoculated with 10^5.1^ TCID_50_ of A(H6N1) or 10^5.2^ TCID_50_ of A(H7N9) virus; both inoculation doses corresponded to ∼5.0 MLD_50_. The experiment with A(H7N9) virus was done in duplicate, using 10 mice per group for the second round. 81.39a (15 or 45 mg/kg) or control IgG (45 mg/kg) was given once at 48 h postinoculation (hpi). Mice infected with A(H6N1) virus were treated only with the higher MAb concentration. Lungs (*n* = 6/treatment group) were collected at days 5 and 7 pi; for H7N9-infected mice, lungs were collected only from the first experiment. Viral titers in lung homogenates were determined using the TCID_50_ assay. Mice were observed daily for clinical signs and survival throughout the study.

### Infection, treatment, and collection of nasal wash, blood, and tissue samples in ferrets.

Groups of four male ferrets (Mustela putorius furo) aged 3 to 5 months (Triple F Farms, PA), and serologically negative by hemagglutination inhibition (HI) assay for currently circulating influenza A(H1N1)pdm09, A(H3N2), and B viruses, were used in this study. Ferrets were housed in customized, individual cages (Allentown Inc., Allentown, PA) and monitored for at least 3 days for acclimation and to establish baseline body temperatures prior to the start of the study. Clinical signs of illness and body weight were recorded daily. The following activity status scores were used: 0, alert and playful; 1, alert and playful only when stimulated; 2, alert but not playful when stimulated; 3, neither alert nor playful when stimulated. Nasal and ocular discharge, sneezing, coughing, dyspnea (shortness of breath), and diarrhea were scored 0 if not present or 1 if present. The temperature was measured twice daily by subcutaneous implantable temperature transponders (Bio Medic Data Systems, Seaford, DE).

Intranasal inoculation was performed under anesthesia, induced by intramuscular administration of a ketamine-xylazine-atropine mixture (25 mg/kg, 2 mg/kg, and 0.05 mg/kg, respectively), using 10^6^ TCID_50_ of virus diluted in sterile phosphate-buffered saline (PBS) (0.5 ml total volume). The A(H1N1)pdm09 virus was used in this study. The infected ferrets were then divided into four groups (four animals per group): control IgG (gD5237; 25 mg/kg) given at 1 dpi and 81.39a (25 mg/kg) given at 24, 48, and 72 hpi. The antibodies (≤1.5 ml total volume) were administered once via intravenous injection through the cranial vena cava.

Nasal washes were collected daily (under anesthesia) by flushing nostrils with 1 ml of sterile PBS and further processed for determination of infectious viral titers using TCID_50_ assay. Under anesthesia, all animals from each group were thoroughly exsanguinated prior to euthanization at 5 dpi, and viral titers were determined in nasal turbinates, trachea, and lung bronchoalveolar lavage fluid (BALF). The BALF was centrifuged at 1,000 × *g* for 10 min, and the inflammatory cell count and protein concentration in cell-free solution were determined. Harvested whole lungs were fixed in 10% neutral buffered formalin and used for histopathology and immunohistochemistry.

### Histopathology and immunohistochemistry.

Histopathology and detection of viral NP in the lung (immunohistochemistry) were performed at the Yerkes National Primate Research Center (Emory University, GA) according to procedures published previously (37). Briefly, the whole lungs were collected immediately after euthanization, fixed by submerging the tissues in 10% neutral buffered formalin, and embedded in paraffin. Five-micrometer sections of each lung lobe were taken and stained with hematoxylin and eosin (H&E). For immunohistochemistry staining, lung sections were stained with multisubtype mouse anti-NP antibody (1:1,000 dilution; MAB8251; Millipore, CA) by incubating them at room temperature for 1 h. The labeled tissue sections were stained with Mouse Mach2 (Biocare Medical), and all reactions were detected by development of the chromogen (Warp Red; Biocare Medical). Appropriate positive and negative controls were run in parallel. The nuclei were then counterstained using Gill's hematoxylin. Pathological scores were determined as described previously (38). Percent lung affected, severity of alveolitis and bronchiolitis/bronchitis, and extent of peribronchial and peribronchiolar/perivascular infiltrates were given the following scores: 0, no lesions; 1, ≤25%; 2, 25 to 50%; 3, >50%. Alveolar edema, hemorrhage, and type 2 pneumocyte hyperplasia were scored 0 if not present or 1 if present.

### Statistical analysis.

Statistics were performed using the GraphPad Prism v.5.0. software, and the statistically significant difference was set at an alpha of 0.05 (*P* < 0.05). The unpaired two-tailed Student's *t* test was applied to evaluate the statistical significance in viral titers and other analyzed parameters of control IgG- and 81.39a-treated animals. The survival fractions were estimated by the Kaplan-Meier method and compared between treatment groups within the same virus strain using the log-rank (Mantel-Cox) test.

## RESULTS

### 81.39a demonstrates broad-spectrum antiviral activity *in vitro*.

To assess the breadth of 81.39a activity against multiple subtypes, we evaluated its ability to neutralize a panel of influenza A viruses of group 1 and 2 HAs isolated between 1960 and 2015 (Table 1). The panel represented influenza viruses of 16 HA and nine NA subtypes and included isolates with known markers of resistance to FDA-approved NAI. The determined EC_50_s varied widely among viruses tested; however, the infectivity of a majority of viruses was neutralized by 50% at concentrations below 5 μg/ml. H5 (0.10 to 0.22 μg/ml) and H4 (<0.01 μg/ml) subtypes were the most sensitive to neutralization in HA groups 1 and 2, respectively. Notably, a total of 6 viruses representing five HA subtypes were not neutralized at 50 μg/ml, the highest concentration of 81.39a tested (Table 1); they were H11, H12, H13, and H16 subtypes of group 1 HA and A(H7N9) of group 2 HA. It is noteworthy that, for A(H7N9), the mean EC_50_ for five other strains tested in the neutralization assays was 1.5 μg/ml, suggesting strain-specific neutralization of MAb 81.39a. Overall, 81.39a neutralized the majority of influenza viruses of group 1 and 2 HAs tested *in vitro*, signifying its broad-spectrum antiviral activity.

**TABLE 1:** Activity of 81.39a against influenza A viruses of group 1 and 2 HAs in cell culture^*a*^

Clade and strain designation	Subtype	Resistance marker^*d*^		81.39a EC_50_^*b*^ (μg/ml)	Amino acid at position HA_2–_19^*c*^	Accession number in GISAID^*e*^		
		NA	M2			HA	NA	M
Group 1 HA								
A/California/12/2012	A(H1N1)pdm09		S31N	1.29 ± 0.23	D	EPI397889	EPI397888	EPI397887
A/Texas/23/2012	A(H1N1)pdm09	H274Y	S31N	0.75 ± 0.24	D	EPI397892	EPI397891	EPI397890
A/Maryland/08/2013	A(H1N1)pdm09		S31N	0.58 ± 0.04	D	EPI507529	EPI507528	EPI508430
A/Louisiana/08/2013	A(H1N1)pdm09	H274Y	S31N	0.59 ± 0.43	D	EPI507531	EPI507530	EPI515738
A/Minnesota/14/2012	A(H1N2)v		S31N	3.01 ± 0.47	D	EPI395306	EPI395305	EPI395302
A/Ann Arbor/6/1960	A(H2N2)			1.36 ± 0.27	D	EPI400083	EPI400085	EPI400084
A/Vietnam/1203/2004	A(H5N1)		L26I and S31N	0.13 ± 0.03	D	EPI123515	EPI116648	EPI25613
A/duck/Vietnam/NCVD-664/2010 (2.3.2.1a)	A(H5N1)	H274Y		0.22 ± 0.05	D	EPI424370	EPI424369	EPI424365
A/duck/Vietnam/NCVD-680/2011 (2.3.2.1a)	A(H5N1)			0.16 ± 0.05	D	EPI424378	EPI424377	EPI424373
A/northern pintail/Washington/40964/2014 (2.3.4.4)	A(H5N2)		S31N	0.22 ± 0.09	D	EPI569383	EPI569385	EPI569386
A/gyrfalcon/Washington/41088–6/2014 (2.3.4.4)	A(H5N8)		S31N	0.10 ± 0.02	D	EPI569390	EPI569392	EPI569393
A/Taiwan/2/2013	A(H6N1)		S31N	1.93 ± 0.89	D	EPI459855	EPI459857	EPI459858
A/garganey/Ukraine/05835-NAMRU3/2006	A(H8N4)			0.31 ± 0.07	D	EPI372512	EPI372511	EPI372507
A/chicken/Vietnam/NCVD-1156/2011	A(H9N2)		S31N	0.08 ± 0.03	**A**	EPI457483	EPI457482	EPI457478
A/chicken/Bangladesh/OP-4/2013	A(H9N2)		S31N	<1.0	**A**	EPI744942	EPI744941	EPI744940
A/environment/Bangladesh/OE-09/2013	A(H9N2)		S31N	<1.0	**A**	EPI744945	EPI744944	EPI744943
A/quail/Bangladesh/337/2013	A(H9N2)			<1.0	**A**	EPI744948	EPI744947	EPI744946
A/Hong Kong/308/2014	A(H9N2)		S31N	0.11 ± 0.02	**A**	EPI498037	EPI498036	EPI498032
A/duck/Bangladesh/1595/2010	A(H11N3)		S31N	>50	**N**	EPI540159	EPI540158	EPI540154
A/mallard/Alberta/60/1976	A(H12N5)			>50	**A**	EPI744935	EPI744934	NS
A/lesser flamingo/Kenya/54/2008	A(H12N2)		Unknown	>50	**A**	EPI744937	EPI744936	NS
A/shorebird/Delaware/68/2004	A(H13N9)			>50	**N**	EPI744939	EPI744938	NS
A/shorebird/Delaware/172/2006	A(H16N3)			>50	**N**	EPI407987	EPI407997	EPI407996
Group 2 HA								
A/Wuhan/359/1995-like	A(H3N2)			1.23 ± 0.18	D	EPI828813	EPI828814	NS
A/Wuhan/359/1995-like	A(H3N2)	E119V		1.06 ± 0.11	D	EPI828815	EPI828816	NS
A/Bethesda/956/2006	A(H3N2)		S31N*	4.85 ± 0.31	D	EPI746386	EPI244127	NS
A/Texas/12/2007	A(H3N2)	E119V	S31N	0.84 ± 0.11	D	EPI466983	EPI466982	EPI466981
A/Ohio/88/2012	A(H3N2)v	S247P	S31N	2.83 ± 0.83	D	EPI397961	EPI397960	EPI397957
A/turkey/Minnesota/833/1980 (clone 1)	A(H4N2)	R292K*		<0.01	D	EPI240428	NS	EPI240272
A/turkey/Minnesota/833/1980 (clone 2)	A(H4N2)			<0.01	D	EPI243157	EPI243159	EPI243160
A/turkey/Indiana/1573–02/2016	A(H7N8)			0.15 ± 0.01	D	EPI744951	EPI744950	EPI744949
A/turkey/Indiana/1403/2016	A(H7N8)			0.28 ± 0.02	D	EPI744954	EPI744953	EPI744952
A/Anhui/1/2013	A(H7N9)			3.93 ± 1.85	D	EPI439507	EPI439509	EPI439506
A/Shanghai/1/2013 (clone 27)	A(H7N9)		S31N	0.3 ± 0.19	D	EPI744956	EPI503954	EPI744955
A/Shanghai/1/2013 (clone 1)	A(H7N9)	R292K	S31N	0.66 ± 0.12	D	EPI439486	EPI439487	EPI439493
A/Taiwan/01/2013 (clone 33)	A(H7N9)		S31N	0.83 ± 0.25	D	EPI515454	EPI503953	EPI515448
A/Taiwan/01/2013 (clone S2)	A(H7N9)	R292K	S31N	>50	**G**	EPI516362	EPI503956	EPI516364
A/Hong Kong/734/2014	A(H7N9)		S31N	1.71 ± 0.19	D	EPI498800	EPI498799	EPI498795
A/duck/Vietnam/NCVD-0035/2012	A(H10N7)			0.43 ± 0.1	D	EPI744933	EPI744932	EPI744931
A/garganey/Ukraine/05839-NAMRU3/2006	A(H14N6)			0.14 ± 0.02	D	EPI372520	EPI372519	EPI372515
A/shearwater/West Australia/2576/1979	A(H15N9)		Unknown	0.61 ± 0.23	D	EPI184623	EPI184640	NS

a The EC_50_ data were generated in neutralization assays. The 81.39a antibody was present throughout viral replication cycles. Influenza B virus-specific antibody was used as an IgG control.

b Each data point ± standard deviation represents the mean EC_50_ done in triplicate.

c The HA_2_-D19A/G/N amino acid substitutions are shown in boldface.

d Asterisks indicate molecular markers of resistance to NAIs (NA) and/or M2 inhibitors (M2) as determined using pyrosequencing assay.

e NS, not sequenced.

### Sequence analysis of viruses with reduced *in vitro* susceptibility.

The observed subtype- and strain-specific neutralization pattern prompted further investigation to identify a molecular basis for the reduced neutralizing activity. As 81.39a has predicated binding to the HA stalk region (26), we analyzed HA sequences of all tested viruses and aligned sequences within the stalk region to examine amino acid differences. Our analysis showed that all six viruses that were not neutralized shared a substitution at position 19 in HA_2_ (HA_2_ numbering starting from the HA_0_ cleavage site) (Table 1). Specifically, HA_2_-Asp19Asn was found in H11, H13, and H16 subtype viruses; these three subtypes form cluster H1b of group 1 (39, 40). Two H12 subtype viruses tested had HA_2_-Asp19Ala; this subtype belongs to cluster H9 of group 1 HA (40). An A(H7N9) virus, A/Taiwan/1/2013 (clone S2), possessed HA_2_-Asp19Gly (Table 1).

We next compared the 81.39a neutralization potency against two clones of A/Taiwan/01/2013 [A(H7N9)] influenza virus that differed by a single amino acid substitution in HA_2_ using a focus-forming reduction assay (Table 2). While clone C7 possessed HA_2_-Asp19 (wild type), clone S2 possessed HA_2_-Asp19Gly. In the first set of experiments, the maximum MAb concentration tested was 50 μg/ml. As shown in Table 2, 50% of the HA_2_-Asp19 clone virus was inhibited by 3.55 μg/ml of 81.39a (61% reduction in focus number; range, 47 to 76%). The 50 μg/ml concentration of MAb failed to reach the 50% (32% reduction in focus number; range, 19 to 44%) infectivity inhibition for the HA_2_-Asp19Gly clone compared to that of the control (infected, untreated cells). In the second set of experiments the maximal concentration of 81.39a was increased to 300 μg/ml. We found that the number of foci produced by the HA_2_-Asp19Gly clone in cells treated with 300 μg/ml of 81.39a was much lower. Approximately 48% inhibition (range, 22% to 54%) was observed. The results confirmed that the HA_2_-Asp19Gly substitution reduced the ability of the MAb to neutralize virus infectivity *in vitro*.

**TABLE 2:** Neutralization activity of 81.39a against A(H7N9) viruses^*a*^

Expt no. and H7N9 virus	Amino acid at HA_2–_19	No. of foci at the indicated concertation of 81.39a^*b*^ (% inhibition; range)			EC_50_ (μg/ml)
		0 μg/ml	50 μg/ml	300 μg/ml	
1					
A/Taiwan/01/2013 (clone C7)	D	43.7 ± 5.4	17.0 ± 3.6 (61; 47–76)	NT	3.55 ± 0.85
A/Taiwan/01/2013 (clone S2)	G	45.7 ± 2.6	31.3 ± 3.1 (32; 19–44)	NT	>50
2					
A/Taiwan/01/2013 (clone C7)	D	48.0 ± 10.2	15.7 ± 3.7 (67; 47–82)	8.3 ± 1.7 (83; 74–90)	3.06 ± 0.63
A/Taiwan/01/2013 (clone S2)	G	51.3 ± 2.1	43.3 ± 2.1 (16; 6–24)	32.0 ± 5.4 (48; 22–54)	≥300

a Virus was preincubated for 1 h with 81.39a, followed by 1 h of virus adsorption. The MAb was present in cell overlay throughout the experiment.

b Mean number of foci derived from at least three replicates for each virus per experiment. Values are compared to those in untreated (0 μg/ml) cells. NT, not tested.

### Polymorphism frequencies at position HA_2_-Asp19 among influenza A viruses.

To understand the frequency of amino acid polymorphisms at residue 19 in the HA_2_ protein, we examined HA sequences deposited in GISAID as of 12 November 2015. The majority (∼99%) of H7N9 viruses recovered from humans (*n* = 231) maintained HA_2_-Asp19; only two viruses, A/Jiangsu/02/2013 and A/Wuxi/1/2013, contained Asp19Asn, and a single virus, A/Shenzhen/SP39/2014, had Asp19Gly. Conversely, all HA sequences of H11 (*n* = 619), H13 (*n* = 176), and H16 (*n* = 58) viruses possessed Asn, while all H12 subtype sequences (*n* = 150) possessed Ala at this position. Peculiarly, while the H12 subtype belongs to the H9 cluster of group 1 HA, the presence of Ala19 in the A(H9N2) viruses tested had no effect on virus neutralization (Table 1). The majority (∼99%) of H9 subtype viruses recovered from humans and other species possessed Ala19 (over 3,000 HA sequences).

### Therapeutic efficacy of 81.39a against group 1 HA influenza A viruses in mice.

**FIG 1:**
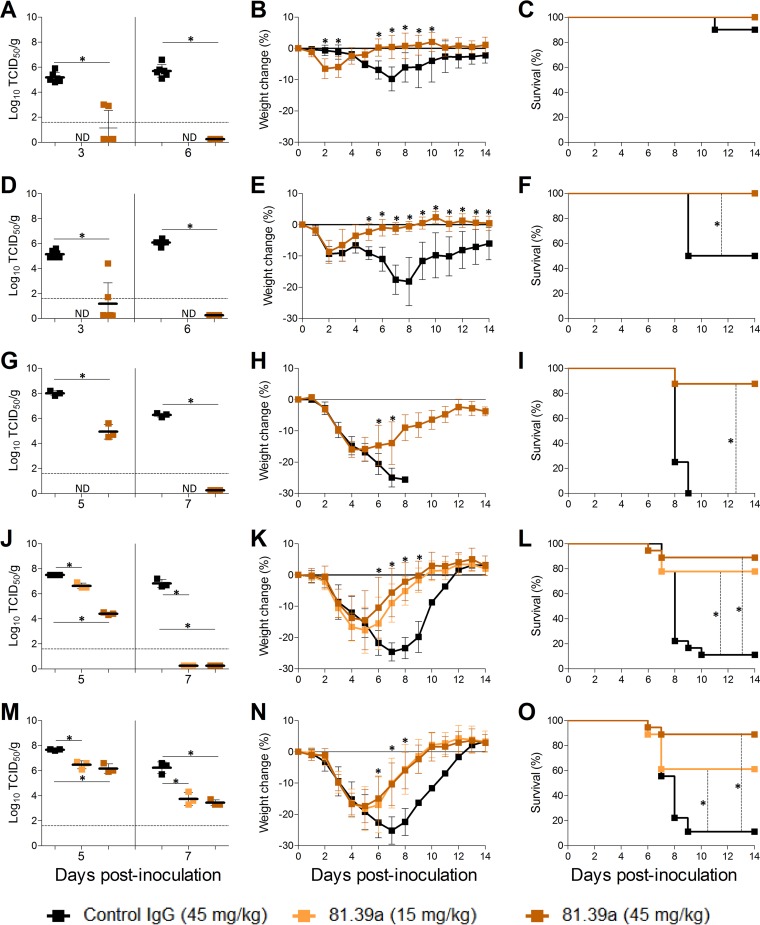
Effectiveness of 81.39a treatment in mice. Viral titers in lungs (A, D, G, J, and M), change in body weight (B, E, H, K, and N), and survival (C, F, I, L, and O) of mice inoculated with 10^5.6^ EID_50_ of H5N2 (A to C), 10^6.6^ EID_50_ of H5N8 (D to F), 10^5.1^ TCID_50_ of H6N1 (G to I), 10^5.2^ TCID_50_ of A(H7N9) wild-type (J to L), or 10^5.2^ TCID_50_ of A(H7N9) HA_2_-Asp19Gly (M to O) virus and treated at 24 hpi for A(H5N2), A(H5N8), and A(H6N1) or 48 hpi for A(H7N9) with 81.39a at the indicated dose. All data are expressed as the means ± SD. (A and B) Representative results from an experiment with 22 mice per group. (C) Representative results from an experiment with 14 mice per group. (D and E) Representative results from two independent experiments with 10 to 14 mice per group. Dotted lines in lung viral titers indicate the limit of detection (<1.6 log_10_). *, *P* < 0.05; ND, not determined.

Because *in vitro* neutralization is not always predictive of *in vivo* effectiveness of influenza antivirals, we tested the efficacy of 81.39a against emerging zoonotic influenza A viruses. First, we evaluated the therapeutic efficacy of 81.39a against HPAI A(H5) viruses as a representative of group 1 HA viruses. Mice were inoculated with either an A(H5N2) (A/northern pintail/Washington/40964/2014) or A(H5N8) (A/gyrfalcon/Washington/41088-6/2014) influenza virus and given a single intravenous dose of 81.39a (45 mg/kg) at 24 hpi. 81.39a administration protected 100% of animals from death (Fig. 1C and F). Of note, in the control IgG (gD5237)-treated groups, 90% of A(H5N2)-inoculated and 50% of A(H5N8)-inoculated animals survived the infection, confirming previous results that recent clade 2.3.4.4 A(H5N2) and A(H5N8) viruses detected in North America demonstrate a moderate pathogenicity phenotype in mice (36) compared to other Eurasian-lineage HPAI A(H5) viruses. Both A(H5N2) and A(H5N8) viruses replicated efficiently in the lungs of control mice at 3 and 6 dpi; their mean titers ranged from 5.2 to 6.1 log_10_ (Fig. 1A and D). Treatment with 81.39a significantly reduced mean viral titers in the lungs up to >3 log_10_ at day 3 (*P* < 0.0001 for both viruses) and virus was not detected (>5 log_10_ reduction) at day 6 (*P* < 0.0001 for both viruses), indicating that 81.39a controlled virus spread in the lower respiratory tract of mice. Further, 81.39a treatment prevented body weight loss (Fig. 1B and E), and 81.39a-treated mice recovered much faster from the disease than those in the control groups.

The 81.39a efficacy against another group 1 HA virus (A/Taiwan/2/2013) of A(H6N1) subtype was also tested in mice. In this experiment, the same dose (45 mg/kg) of antibody as that used for H5-infected animals was administered at 48 hpi. As expected, control IgG-treated animals exhibited more severe signs of disease and succumbed to infection between 8 and 9 dpi (Fig. 1I). Conversely, 81.39a treatment protected ∼90% of infected animals and prevented significant weight loss (Fig. 1H). Compared to the control group, 81.39a significantly reduced mean viral titers in the lungs of A(H6N1)-inoculated mice at 5 dpi (>3 log_10_; *P* < 0.0001); no virus was detected at 7 dpi (>5 log_10_ reduction; *P* < 0.0001) (Fig. 1G). Altogether, these data show that 81.39a was effective in mice infected with a group 1 HA influenza virus.

### Therapeutic efficacy of 81.39a against group 2 HA influenza A virus in mice.

Antiviral efficacy of 81.39a against group 2 influenza virus was evaluated in the A(H7N9) lethal model. Two doses, 15 and 45 mg/kg, were given to animals at 48 hpi. Viral titers in lungs were determined at 5 and 7 dpi, and morbidity and mortality were monitored for 14 days. To maximize the translational relevance, we used both HA_2_-Asp19 (wild type) and HA_2_-Asp19Gly A(H7N9) (A/Taiwan/1/2013) influenza viruses in our experiments.

The experiment with each virus was performed in duplicate. Both viruses replicated to comparably high titers in lungs of mice (Fig. 1J and M). Moreover, they showed a similar level of virulence, as judged by body weight loss and survival (Fig. 1K, L, N, and O). Treatment of wild-type-infected mice with 81.39a resulted in a dose-dependent inhibition in viral titers in lungs at 5 dpi with mean titers reduced by 0.9 log_10_ (*P* = 0.004) and 3.1 log_10_ (*P* < 0.0001) for lower and higher doses, respectively (Fig. 1J). At day 7, viral titers in lungs remained high (6.8 log_10_) in control IgG-treated mice, while no virus was detected in animals treated with either dose of 81.39a (Fig. 1J), indicating a strong antiviral effect despite the delayed treatment.

Infection with either of the A(H7N9) viruses caused ∼90% mortality in control IgG-treated animals (Fig. 1L and O). The lower and higher 81.39a doses protected 14 of 18 (∼80%; *P* = 0.0007) and 16 of 18 (∼90%; *P* < 0.0001) mice inoculated with the wild-type virus (Fig. 1L). In mice inoculated with the HA_2_-Asp19Gly virus, the higher dose of antibody resulted in protection of 16 of 18 (∼90%, *P* < 0.0001) mice and the lower dose protected 11 of 18 (∼60%; *P* = 0.013) (Fig. 1O). Therefore, the diminishing effect of the HA_2_-Asp19Gly substitution on survival was only observed at the lower dose of treatment.

The inhibitory effect of 81.39a on virus replication was also observed in lungs of mice inoculated with the HA_2_-Asp19Gly A(H7N9) virus. The lower and higher doses reduced mean lung viral titers by 1.2 log_10_ (*P* = 0.002) and 1.5 log_10_ (*P* = 0.003) at day 5, respectively (Fig. 1M). At 7 dpi, control IgG-treated mice had high viral titers (6.2 log_10_), while those that received 81.39a had significantly lower lung viral titers, 3.7 log_10_ with 15 mg/kg (*P* = 0.004) and 3.4 log_10_ with 45 mg/kg (*P* = 0.001). Overall, the antiviral effect of 81.39a was less pronounced in the HA_2_-Asp19Gly-infected group than in the mice inoculated with the wild-type A(H7N9) virus.

Other clinical indices mirrored the antiviral effect observed in the lungs of 81.39a-treated mice. Disease manifestations such as lethargy, ruffled fur, hunched posture, and dyspnea were apparent in control groups infected by either H7N9 virus but were rarely to occasionally present in the majority of 81.39a-treated animals. The body weight loss in 81.39a-treated animals was less pronounced and their recovery occurred much faster than that of the control groups (Fig. 1K and N). The dose-dependent effect of treatment was not apparent, particularly in mice infected with the HA_2_-Asp19Gly virus.

Overall, these data indicate that 81.39a was also effective in mice infected with a group 2 HA influenza virus, demonstrating that *in vitro* antiviral activity is not always a predictive factor of efficacy in mammalian animal models.

### 81.39a efficacy against A(H1N1)pdm09 influenza virus in ferrets.

We next utilized a ferret model to assess the effect of 81.39a treatment on viral replication in the upper and lower respiratory tracts. A/California/12/2012, a representative of an A(H1N1)pdm09 virus, was used to analyze 81.39a effectiveness in ferrets. A single intravenous treatment was given to ferrets at 24, 48, or 72 hpi to assess the effect of delayed treatment. Either 81.39a or control IgG was delivered at a dose of 25 mg/kg. Nasal wash samples were collected from 1 through 5 dpi before animals were humanely sacrificed to harvest respiratory organs.

Infection of ferrets with the A(H1N1)pdm09 virus caused moderate to severe signs of disease (Table 3). All inoculated animals showed ∼5 to 7% body weight loss and had nasal discharge, indicative of productive infection. The number of 81.39a-treated animals that developed fever and demonstrated clinical symptoms, namely, sneezing, cough, and labored breathing, was substantially lower than that of control IgG-treated groups. However, regardless of treatment, none of these animals had blood oxygen levels (i.e., saturation of peripheral oxygen [SpO_2_]) below 90%, which, if present, would indicate hypoxemia and/or respiratory failure.

**TABLE 3:** Clinical signs in A(H1N1)pdm09-infected ferrets treated with or without 81.39a^*a*^

Parameter	Value by time of treatment (hpi) for:			
	Control IgG (24 hpi)	81.39a		
		24	48	72
No. positive/total no.				
Weight loss (% of maximum)	4/4 (6)	4/4 (6)	4/4 (5)	4/4 (7)
Pyrexia^*b*^	3/4	2/4	1/4	2/4
Sneeze (frequency)	4/4 (high)	4/4 (low)	4/4 (moderate)	4/4 (moderate)
Nasal discharge	4/4	4/4	4/4	3/4
Cough	2/4	0/4	0/4	0/4
Dyspnea	2/4	0/4	0/4	0/4
SpO_2_^*c*^ (%)	95–100	95–100	96–100	95–100

a Ferrets (*n* = 4 per group) were infected with 10^6^ TCID_50_ of A(H1N1)pdm09 virus and treated with 25 mg/kg of control IgG (gD5237) or 81.39a at 24, 48, or 72 hpi.

b Increase in temperature of ≥1.5°C compared to the baseline.

c Saturation of peripheral oxygen (SpO_2_), measured during days 2 to 5 postinoculation.

**FIG 2:**
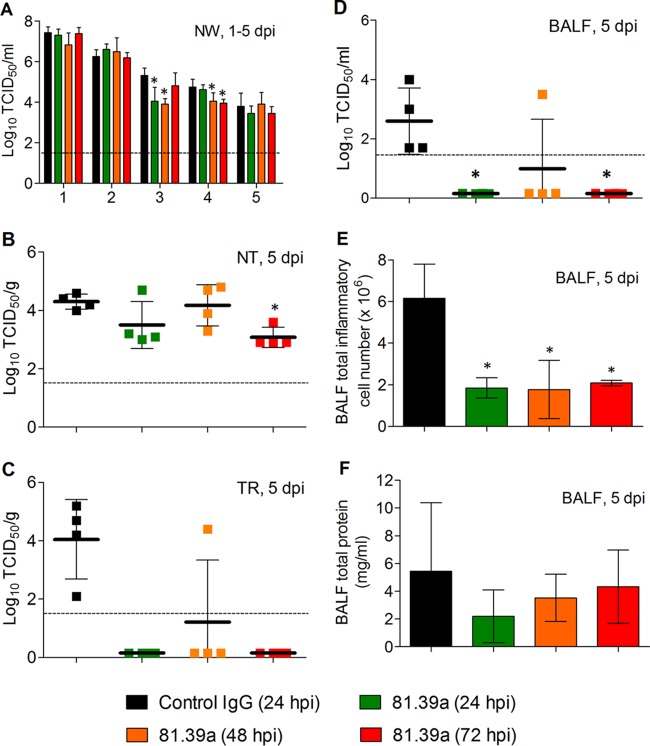
Effectiveness of 81.39a treatment in ferrets. Viral titers in nasal washes (NW) (A), nasal turbinates (NT) (B), trachea (TR) (C), and bronchoalveolar lavage fluid (BALF) (D), as well as inflammatory cell counts (E) and protein concentrations (F) in BALF. Groups of four ferrets were inoculated with 10^6^ TCID_50_ of H1N1pdm virus and treated with 25 mg/kg of control IgG or 81.39a at the indicated hours postinoculation. Nasal washes were collected 1 to 5 dpi, while respiratory tract tissues and BALF were harvested at 5 dpi. All data are expressed as the means ± SD. In panels B to D, each data point represents the viral titer of an individual animal. Dotted lines in lung viral titers indicate the limit of detection (<1.6 log_10_). *, *P* < 0.05.

All inoculated animals shed virus throughout the 5-day experiment (Fig. 2A); nasal wash viral titers peaked at day 1 (∼7 log_10_), which is not an unusual phenomenon when a high dose (10^6^ TCID_50_) of virus is used for inoculation. The 24-h delayed treatment with 81.39a resulted in a significant viral titer reduction (1.3 log_10_; *P* = 0.016) detected at 3 dpi. When given at 48 hpi, 81.39a treatment significantly reduced nasal wash viral titer at 3 (1.4 log_10_; *P* = 0.0007) and 4 (0.7 log_10_; *P* = 0.046) dpi. Notably, a statistically significant difference in viral titer reduction was also detected at 4 dpi, even when 81.39a treatment was delayed for 72 h (0.7 log_10_; *P* = 0.009).

The A(H1N1)pdm09 influenza virus replicated in the lung tissues, consistent with previous reports (37, 41, 42). This virus replicated to relatively high titer (∼4 log_10_) not only in the nasal turbinate but also in the trachea and, to some extent, in bronchoalveolar lavage fluid (BALF) of control IgG-treated ferrets (Fig. 2B to D) at 5 dpi. When administered at 72 hpi, 81.39a produced a noticeable reduction in viral titers (1.2 log_10_; *P* = 0.0013) in the nasal turbinate (Fig. 2A); these changes were not statistically significant for 24- or 48-h-delayed treatment. A more pronounced effect from 81.39a treatment was found in the trachea and BALF samples (Fig. 2B and C). Virus was not detected in any of these samples collected from animals treated with 81.39a at 24 or 72 hpi, and only one of four ferrets treated with 81.39a at 48 hpi had detectable viral titers in the trachea (*P* = 0.01 for both time points) and BALF (*P* = 0.005 for both time points). The results suggest a superior antiviral activity of 81.39a in the lower respiratory tract compared to the upper respiratory tract.

Subsequently, we measured the total inflammatory cell number and protein content in the BALF of ferret at 5 dpi. BALF total cell numbers were decreased significantly after 81.39a treatment given at 24 (*P* = 0.004), 48 (*P* = 0.006), or 72 (*P* = 0.008) hpi (Fig. 2D). Although not statistically significant, a trend toward lower BALF total protein content was found across all 81.39a-treated groups (Fig. 2E), suggesting that the 81.39a treatment was beneficial in protecting pulmonary tissues from the severe damage that occurred during viral infections of control IgG-treated ferrets.

### 81.39a administration alleviates pathological changes in the lungs of ferret.

**FIG 3:**
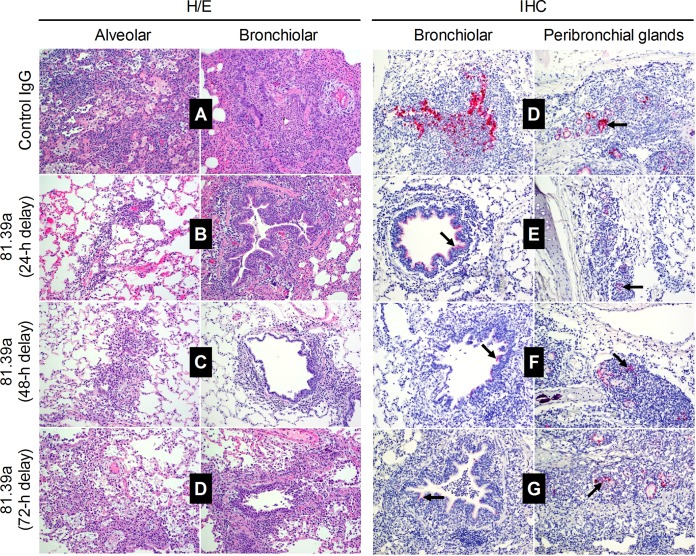
Pulmonary histopathology lesions and antigen expression in infected ferrets. Animals were inoculated with 10^6^ TCID_50_ of A(H1N1)pdm09 virus and treated with 25 mg/kg of control IgG or 25 mg/kg of 81.39a at 24, 48, or 72 hpi. Lungs were collected from each animal at day 5. Each image was derived from one sample as a representative of the respective treatment group with four ferrets per group. Viral NP stains are shown by black arrows. Images were taken with ×200 magnification. H/E, hematoxylin and eosin stain; IHC, immunohistochemistry.

To further investigate the therapeutic effect of 81.39a in the lower respiratory tract of ferrets, we examined the pulmonary histopathology and the presence of viral NP in the lung sections of each lobe of virus-inoculated animals. Lung tissues of control IgG-treated ferrets showed severe multifocal alveolar, bronchial, perivascular, and interstitial infiltrates of inflammatory cells composed of neutrophils, macrophages, and a few lymphocytes intermixed with occasional glandular and lymphoid necrosis, fibrin deposition, and edema (Fig. 3A and Table 4). Congested alveolar walls were found frequently and contained type II pneumocyte hyperplasia or infiltrates of lymphocytes and macrophages. The bronchial and bronchiolar epithelium was necrotic and mildly hyperplastic. Overall, the pathology damage in lung samples of control animals was assessed as severe (Table 4). In contrast, lung samples from 81.39a-treated animals (24- and 48-h-delayed treatment) displayed moderate degrees of tissue damage (Fig. 3B and C and Table 4). 81.39a also prevented severe lesions in the lungs when given 72 hpi (Fig. 3D), but its therapeutic effect was less significant than that of the 24- and 48-h-delayed treatment. This finding showed that a faster tissue recovery took place in the lungs of 81.39a-treated animals than in those of control IgG-treated animals.

**TABLE 4:** Histologic findings in the respiratory tracts of infected ferrets treated with 81.39a or left untreated

Antiviral and time of treatment (hpi)	Lung tissue analysis^*a*^	
	Histopathological lesions (score ± SD)	Detection of viral NP
Control IgG		
24	Severe (8.5 ± 0.5)	Frequent
81.39a		
24	Moderate (6.3 ± 1.1)	Rare to occasional
48	Moderate (6.3 ± 1.5)	Rare to occasional
72	Moderate to severe (7.0 ± 1.6)	Rare to occasional

a Ferrets were inoculated with 10^6^ TCID_50_ of A(H1N1)pdm09 virus and treated with 25 mg/kg of control IgG (gD5237) at 24 hpi or 81.39a at 24, 48, or 72 hpi. Each data point ± SD represents the mean score of histopathological lesions from 4 animals per group.

To confirm that the observed lung damage was caused by virus replication and to examine the effect of 81.39a administration on the degree of virus replication in the lungs, we stained lung tissue samples for viral NP using immunohistochemistry. The lung sections derived from control animals showed frequent NP staining in the bronchiolar and peribronchial lymphoid and glandular tissues (Fig. 3D and Table 4). Conversely, the number of positively NP-stained cells was markedly lower in 81.39a-treated animals in all regimens tested. Detection of viral NP in the lungs was rare or only occasional in animals receiving 24-, 48-, or 72-h-delayed treatment (Fig. 3E to G and Table 4). These data suggest that 81.39a treatment contributed to rapid elimination of viruses from the lower respiratory tract of virus-inoculated animals.

## DISCUSSION

Here, we demonstrate the broad neutralization activity of human MAb 81.39a, which targets influenza A viruses of HA groups 1 and 2, using a comprehensive panel of influenza A viruses encompassing 16 HA subtypes (23 viruses from group 1 and 17 from group 2). This panel contained viruses collected from various geographic locations and included seasonal viruses, zoonotic viruses, HPAI A(H5N2) and A(H5N8) viruses, subtypes responsible for an unprecedented outbreak in poultry in the U.S., and virus variants resistant to one or both classes of FDA-approved anti-influenza drugs. The strength of this study is the direct testing of this diverse group of viruses using the same *in vitro* neutralization method, while previous studies on group 1- and 2-targeting MAbs only tested selected subtypes in the neutralization assay (21–25). In addition, conclusions of the reported subtype coverage were made based on the antibody binding to recombinant proteins or results from neutralization assays using pseudotyped viruses. It is noteworthy that the ability of bnAbs to bind to different HA subtypes does not always prove that they also neutralize virus infectivity, as shown with FJ76 (22). This phenomenon might not be uncommon for HA stalk-targeting antibodies. Factors which could influence the neutralization ability of an antibody include the strength of binding affinity and the location of binding sites within the HA stalk.

The EC_50_s ranged widely (≥500-fold), from <0.01 to 4.85 μg/ml, and there was no consistent difference between group 1 and group 2 viruses. The EC_50_s determined in our study were similar to those reported for viruses tested with the parental MAb 81.39 (0.65 to 26.3 nM, corresponding to 0.10 to 4.11 μg/ml) (21). Several mechanisms of *in vitro* neutralization by MAbs were previously demonstrated, such as prevention of cleavage by cellular protease needed for HA maturation, interference with the fusion between viral and endosomal membranes via binding to a monomeric HA or cross-linking the neighboring HA molecules, and potential disruption of viral egress (22, 25, 43). Accessibility to the antibody epitope seems to be favored when HA molecules are expressed on the surface of infected cells rather than on the surface of the virion (22). Indeed, in our study, virus preincubation with 81.39a had a negligible effect on EC_50_s. In contrast, the impact of the MAb's presence during viral replication steps in cells, where it can bind to newly synthesized HA molecules, was evident (Table 5). Taken together, these results are consistent with the proposed dual effect of bnAbs on virus infection: binding to HA prevents the early stage of viral replication, most likely by interference with fusion, and later, binding to HA expressed on the surface of infected cells disrupts the formation of infectious particles and their spread to neighboring cells (22).

**TABLE 5:** Neutralization activity of 81.39a added at different steps of viral replication

No.	Virus	HA subtype	NA substitution	EC_50_, μg/ml (*n*-fold), for step^*a*^:		
				A^*b*^	B^*c*^	C^*d*^
1	A/California/12/2012	A(H1N1)pdm09		1.29 ± 0.23	3.24 ± 1.09 (3)	NT
2	A/Texas/23/2012	A(H1N1)pdm09	H274Y	0.75 ± 0.24	5.22 ± 3.84 (7)	NT
3	A/Maryland/08/2013	A(H1N1)pdm09		0.58 ± 0.04	10.09 ± 8.72 (17)	0.54 ± 0.22 (1)
4	A/Louisiana/08/2013	A(H1N1)pdm09	H274Y	0.59 ± 0.43	10.02 ± 2.62 (17)	0.65 ± 0.26 (1)
5	A/Wuhan/359/1995-like	A(H3N2)		1.23 ± 0.18	12.92 ± 3.67 (11)	NT
6	A/Wuhan/359/1995-like	A(H3N2)	E119V	1.06 ± 0.11	9.94 ± 1.66 (9)	NT

a EC_50_s were derived from at least three replicates for each virus per experiment. *n*-fold indicates the fold increase in EC_50_ compared to step A. NT, not tested.

b Virus was preincubated for 1 h with 81.39a, followed by 1 h of virus adsorption. The MAb was present in cell overlay throughout the experiment.

c Virus was preincubated for 1 h with 81.39a, followed by 1 h of virus adsorption. Thereafter, inoculum was removed and cells were washed 2× with PBS; no MAb was in the cell overlay.

d Virus was preincubated for 1 h without 81.39a, followed by 1 h of virus adsorption. The MAb was present in cell overlay throughout the experiment.

In this study, viruses whose neutralization by 81.39a was abrogated in the neutralization assay had Asp at position HA_2_-19 replaced with Asn, Ala, or Gly. All but one virus belong to H11, H12, H13, and H16 subtypes (group 1), which are not known to cause human infections. A single virus from group 2, a drug-resistant A(H7N9) clone, had HA_2_-Asp19Gly. It is noteworthy that substitutions at this position are rarely found among A(H7N9) virus recovered from humans. The HA_2_-Asp19Gly virus escaped neutralization by 81.39a at a concentration of 50 μg/ml. However, when the MAb concentration was increased to 300 μg/ml, some degree of inhibition of virus infectivity was observed, suggesting a therapeutic benefit of higher drug doses. It is worth noting that while group 1-targeting MAbs D8 and F10 effectively neutralized a pseudotyped virus of H11 subtype carrying Asp19Asn (40), a lack of neutralization of H3 and H7 subtype viruses carrying this change was reported for CR8020, a group 2-neutralizing MAb (44). The lack of neutralizing activity toward viruses with Asp19 substitutions may be explained by previous structural studies. For instance, residue Asp19 was shown to lie within the epitope, recognized by the light chain of 39.29, which competes with 81.39 for overlapping regions (21). Therefore, a substitution at Asp19 may also affect the binding affinity of 81.39a under our *in vitro* neutralization assay conditions. Further, Asp19Asn was demonstrated to abrogate neutralization of A(H3N2) and A(H7N7) viruses by CR8020 (44), where HA_2_-Asp19Asn is believed to disrupt a possible salt bridge to V_L_ of arginine at position 53, leading to destabilization of the antibody-HA interaction.

Peculiarly, for the group 1- and 2-targeting MAb CR9114, the effect of substitution HA2-Asp19Asn on binding affinity varied among HA subtypes. Asp19Asn reduced the binding affinity (*K_d_* [dissociation constant] ≥ 570 nM) in the H3 subtype, while the binding affinity of the H13 (*K_d_* = 6.3 nM) and H16 (*K_d_* = 20 nM) subtypes carrying HA2-Asn19 was similar to that of the H3 subtype carrying HA2-Asp19 (*K_d_* = 33 nM) (23). Moreover, binding to H9 and H12 subtypes carrying HA_2_-Asp19Ala was not affected (*K_d_* of 2 and 2.9, respectively), similar to that of the H1 subtype. The H9 and H12 subtypes are phylogenetically closely related; H12 belongs to the H9 cluster of group 1 HA (39, 40). In our study, however, the effect of HA_2_-Asp19Ala on virus neutralization by 81.39a was different, indicating the distinctive features of each stalk-binding MAb, and underscores the importance of actual testing of viruses in neutralization assays. Because avian A(H9N2) viruses are known to infect humans (45, 46), there is added utility in demonstrating the ability of 81.39a to neutralize these viruses.

In the mouse model, we used four zoonotic viruses, HPAI A(H5N2), A(H5N8), A(H6N1), and A(H7N9), which showed similar EC_50_s in the neutralization assay. Both H5Nx viruses used in this study have been shown to efficiently replicate in the lungs of mice without prior adaptation (36). Regardless of virus used, a single dose of 1-day-delayed treatment with 81.39a protected animals from weight loss, rescued 90% of animals from the lethal challenge, and resulted in an undetectable level of virus titers in the inoculated animals at 6 dpi. Further, even the single, 2-day-delayed treatment showed significant reduction in viral titers in lungs of mice inoculated with either A(H6N1) or A(H7N9) virus. Each virus was cleared by 7 dpi, and mice survived the lethal challenge, while 90 to 100% of control IgG-treated animals succumbed to infection. The *in vivo* effectiveness of delayed treatment using group 1- and 2-targeting bnAbs, such as 39.29, 81.39, FI6, FI6v3, CT149, 045-051310-2B06, S6-B01, 07-5E01, 41-5D06, and 41-5E04, was also demonstrated in animal models (21, 22, 25, 28, 30).

Interestingly, we observed the therapeutic benefit from 81.39a treatment in mice infected with the A(H7N9) virus carrying HA_2_-Asp19Gly despite its lack of neutralization in cell culture. Similarly, the MAbs VIS410 (27) and 1H5 (47), as well as 41-5D06, 07-5E01, and 24-4C01 (30), were also shown to protect mice from lethal infections with H7N9 virus, although it failed to neutralize the virus *in vitro*. Another prime example is CR9114, which binds to influenza B HA and is protective against influenza B challenge; however, this MAb only neutralizes influenza A and not influenza B viruses (23). Therefore, *in vitro* neutralization may not be completely predictive of *in vivo* potency. The results also suggest the involvement of cellular factors *in vivo* that are lacking in the cell culture system. A previous report indicated that interactions between the fragment crystallizable (Fc) region of broadly neutralizing HA stalk-specific antibodies and Fc receptors (FcγRs) for IgG are required for protection against influenza virus *in vivo* (48). In agreement, an Fc mutant of FI6, lacking both complement and FcγR binding, exhibits 60% less protective efficacy in mice (22), strengthening the importance of FcγR binding in mediating *in vivo* efficacy. One or more of these mechanisms could contribute to the therapeutic benefits observed for 81.39a against HA_2_-Asp19Gly-carrying A(H7N9) viruses *in vivo*: antibody-dependent cell-mediated cytotoxicity (ADCC), antibody-dependent cellular phagocytosis (ADCP), antibody-dependent respiratory burst (ADRB) activity, and complement-dependent cytotoxicity (CDC). A similar *in vivo* phenomenon was also observed with MAbs 1H5 and 1H10, despite the lack of remarkable *in vitro* neutralizing activity (47).

In the ferret model, the antiviral effect of delayed 81.39a treatment was analyzed both in the upper and lower respiratory tracts. While viral titers in nasal washes and nasal turbinate were moderately reduced, a stronger inhibition of viral replication was detected in the trachea and BALF after a single-dose administration of 81.39a, indicative of a higher level of 81.39a activity in the lower respiratory tract compared to the upper respiratory tract. This result was further supported by lung tissue analyses showing a strong reduction of viral NP expression upon 81.39a treatment. In addition, histopathological analyses demonstrate that lung tissues of an uninfected ferret treated with 81.39a displayed no significant lesion, similar to what is observed in lung tissues of an uninfected and untreated control animal (data not shown), indicating that treatment with 81.39a exerts no apparent toxicity.

A previous study suggested that IgA is more efficiently transported to mucosal surfaces, while IgG is found predominantly in the lungs of influenza virus-infected animals (49, 50). Therefore, a lesser antiviral effect of IgG in the upper respiratory tract is expected, which may account for a modest reduction in nasal wash viral titers of 81.39a (IgG)-treated ferrets. Consistent with our finding, another study has shown that serum and lung antibody titers of H1 stalk-specific 6F12 (IgG)-treated ferrets were present in relatively high concentrations at 4 dpi, while the nasal wash antibody titer was similar to that of the negative control on day 3 (51). A pool of the IgA and IgG stalk-targeting antibody mixture may therefore improve the overall therapeutic effect by providing a better location-specific antibody distribution both in the upper and lower respiratory tracts.

To the best of our knowledge, this is the first study in which all 16 HA subtypes of influenza A viruses present in aquatic birds and mammals were used to evaluate a group 1- and 2-targeting bnAb in the neutralization assay. Our data demonstrate that treatment administration of 81.39a is effective in protecting mammals from disease caused by emerging influenza viruses, including H5Nx viruses. Collectively, this study revealed experimental evidence of the therapeutic potential of 81.39a and provided a basis for future clinical trials.
